# Efficacy of Various Feed Additives on Performance, Nutrient Digestibility, Bone Quality, Blood Constituents, and Phosphorus Absorption and Utilization of Broiler Chickens Fed Low Phosphorus Diet

**DOI:** 10.3390/ani12141742

**Published:** 2022-07-07

**Authors:** Shaimaa Selim, Nazema S. Abdel-Megeid, Hanem K. Khalifa, Khloud G. Fakiha, Kamlah A. Majrashi, Eman Hussein

**Affiliations:** 1 Department of Nutrition and Clinical Nutrition, Faculty of Veterinary Medicine, University of Menoufia, Shibin El-Kom 32514, Egypt; 2 Department of Cytology and Histology, Faculty of Veterinary Medicine, University of Sadat City, Sadat City 32897, Egypt; nazemah.abdelmageed@vet.usc.edu.eg; 3 Department of Biochemistry and Chemistry of Nutrition, Faculty of Veterinary Medicine, University of Sadat City, Sadat City 32897, Egypt; hanem.khalifa@vet.usc.edu.eg; 4 Department of Biology, College of Science, University of Jeddah, Jeddah 21493, Saudi Arabia; kgfakiha@uj.edu.sa; 5 Biological Sciences Department, College of Science and Arts, King Abdulaziz University, Rabigh 21911, Saudi Arabia; kmajrashi@kau.edu.sa; 6 Department of Poultry and Fish Production, Faculty of Agriculture, University of Menoufia, Shibin El-Kom 32514, Egypt; eman.hussien@agr.menofia.edu.eg

**Keywords:** phosphorus, probiotic, phytase, organic acid, bone mineralization, carcass traits, hormone profile, bone and gut histology, phosphorus transporter genes

## Abstract

**Simple Summary:**

Skeletal disorders and related-welfare complications are an ongoing concern for rapid-growing broiler chickens. Phosphorus is a vital nutrient in the poultry diet, and it is related to bone growth and skeleton rigidity and strength. Poultry utilize approximately 60% of the dietary P, and the residual is excreted and may enter the environment leading to environmental pollution. Consequently, a better understanding of intestinal phosphorus absorption will enable augmenting phosphorus utilization and reducing phosphorus excretion and feed costs in the poultry industry. Therefore, the present trial was designed to assess various supplements on performance, nutrient digestibility, bone physical parameters and mineralization, blood constituents, bone and gut histomorphology, and duodenal phosphorus transporter genes of broiler chickens fed a decreased non-phytate phosphorus. The dietary addition of phytase or probiotic to a low phosphorus diet was beneficial with respect to growth performance and carcass traits. Regarding nutrient digestibility, tibia quality parameters, and gut and bone histomorphology, all three dietary supplements (probiotics, yeast, and fumaric acid) were advantageous, and these improvements were of comparable magnitude to those of the positive control and phytase groups. The up-regulation of duodenal NaP-IIb and PiT-2 genes in the supplemented groups may suggest greater phosphorus availability. It can be concluded that these supplements were beneficial in mitigating the negative effects of phosphorus reduction on the growth performance, health, and bone quality of broilers.

**Abstract:**

The present trial was designed to assess the effect of phytase, multi-strain probiotic, *Saccharomyces cerevisiae*, and fumaric acid on performance, nutrient digestibility, bone physical parameters and mineralization, blood constituents, bone and gut histomorphology, and duodenal phosphorus transporter genes of broiler chickens fed a decreased non-phytate phosphorus (nPP) diet for 5 weeks. A total of 480 broiler chickens were allotted to six dietary groups and eight replicates each: (1) positive control diet with recommended levels of nPP (PC; 0.48, 0.44, and 0.41% in the three feeding phases); (2) negative control diet with a decreased dietary nPP (NC; 0.28, 0.24, and 0.21% in the three feeding phases); (3) NC + 600 FTU/kg phytase (PHY); (4) NC + 0.05% multi-strain probiotic (PRO); (5) NC + 0.2% *Saccharomyces cerevisiae* (SC); and (6) NC + 0.2% fumaric acid. Growth performance data were recorded weekly, and blood sampling was performed at days 21 and 35 of age. Bone quality traits, gut and tibia histology, nutrient digestibility, and intestinal gene expression analyses were conducted at the end of the trial (35 days of age). Final body weight and total gain at day 35 of age of the broiler chickens fed with the PHY, PRO, and SC diets were greater (*p* < 0.01) than in NC, where broilers fed with the PRO and PHY diets had higher values and were similar to that of PC. There was a non-significant variation in the cumulative feed intake among the treatment groups. The PHY and PRO groups had better FCR than the PC group (*p* < 0.05), and FA and SC had an FCR equivalent to that of PC. The PHY and PRO broilers had greater dressing % than the NC group (*p* < 0.05) and even better than PC. The PHY, PRO, SC, and FA broilers had higher relative weights of spleen and bursa of Fabricius (*p* < 0.01) than NC. In comparison to NC, the PHY, PRO, and SC groups improved (*p* < 0.05) CP, CF, Ca, and P digestibility. Greater tibia breaking strength of the low nPP-supplemented groups was shown to be associated with higher tibia ash, Ca, and P concentrations (*p* < 0.01) and increased (*p* < 0.001) tibia cortical area thickness. At days 21 and 35 of age, the dietary supplements to low nPP diets reduced (*p* < 0.05) serum total cholesterol, triglyceride, triiodothyronine, thyroxine, glucose, and alkaline phosphatase levels, while serum Ca and P concentrations were improved (*p* < 0.05) compared to NC. All supplements led to enhancement (*p* < 0.01) in villi height and width and villi absorptive surface area when compared with NC and were even comparable to that of PC. The mRNA expression of NaP-IIb was up-regulated (*p* < 0.001) in the duodenum of PRO and FA broilers at day 35 of age compared with NC, and their expression levels were similar to that of PC, indicating greater P availability. It is concluded that dietary supplementation of PHY, PRO, SC, and FA to a low nPP diet was advantageous and mitigated the negative impacts of P reduction on the growth performance, health, nutrient digestibility, and bone quality of broilers.

## 1. Introduction

Skeletal problems and related-welfare complications are an ongoing concern for fast-growing chickens and are of great interest in poultry manufacturing [1]. Phosphorus (P) is a vital nutrient in the poultry diet, the second-most essential macro mineral relating to bone growth, and is accountable for skeleton rigidity and strength. Indeed, P shortage or imbalance in broiler diets, particularly during the early and rapid growth stages, cause growth and locomotor complications [2]. Poultry utilize approximately 60% of the dietary P, and the residual is excreted and may induce eutrophication of water systems and environmental P pollution [3]. Consequently, a better analysis of gastrointestinal P absorption will increase P utilization and reduce P waste through excretion. Additionally, P is the third-most-expensive ingredient after energy and protein. Consequently, reducing excess inorganic P in the broiler’s diet will lower the environmental influence of the poultry industry and, additionally, favorably impact the cost of feed [4].

Maize and soybean meal (SBM), the traditional feedstuffs used in poultry diets, contain approximately 8.0–9.0 g of phytate per kg of feed [5] and the majority of P in these feedstuffs is in the phytate form, which is difficult for poultry to utilize due to their low digestive phytase activity. Phytate is a strong chelator of protein and minerals, and its breakdown is essential in the metabolic pathways of birds [6,7]. Therefore, commercial exogenous phytase has been commonly applied in poultry diets to reduce the potential cost and environmental influence triggered by intensive poultry production and enhance the bird’s performance through the superior use of dietary P [6]. The supplementation of phytase is reportedly advantageous for nutrient digestibility and bone mineralization of broilers fed a reduced non-phytate P (nPP) diet [8,9,10]. El-Sherbiny et al. [11] and Nari and Ghasemi [10] observed that the addition of 500 FTU/kg of phytase to low nPP diets (a 0.1% reduction of nPP) enhanced bird performance, nutrient digestibility, and tibia mineralization. Shang et al. [8] reported that the addition of 500 FTU phytase/kg to the diet with a 0.2% reduction in Ca and nPP augmented production, bone parameters, and P utilization in broiler chickens. It is documented that a 0.1 and 0.2% decrease in dietary nPP could be accomplished by phytase addition [8,9,10,11]. However, phytase supplementation has limitations in poultry diets due to its heat sensitivity and cost [12]. Thus, additional strategies are required to encourage P utilization in poultry.

Various approaches were suggested to enhance phytate P availability in the diets of broilers. Organic acids were documented to enhance phytate P utilization in poultry, primarily due to their capacity to reduce the gut pH as a good environment to degrade phytate and decrease the formation of insoluble Ca–phytate complexes [10,13,14]. Organic acids can increase P utilization by broilers fed P-deficient diets [13]. Although the addition of organic acids in the poultry diets was principally aimed to improve production, mineral absorption, gut morphology, and microbiota, it was suggested that organic acids may be accountable for decreased P excretion with valuable effects on the environment [15]. To some extent, phytase is endogenously supplied by the intestinal epithelium and gut microorganisms. Various microorganisms, including bacteria, yeast, and fungi, produce various kinds of enzymes, in particular, phytase [16]. Some microorganisms, such as *Lactobacillus* spp. [17,18,19], *Enterococcus faecium* [20], *Bacillus subtilis* [19,21], and *Aspergillus awamori* [22], were shown to have phytase-like activity and facilitate the utilization of P, enhance intestinal absorption of P, improve bone quality and tibia P content, and decrease P excretion. Additionally, these microbial strains can stimulate growth, augment gut histology, enhance nutrient digestion and absorption, and positively modulate gastrointestinal microbiota in chickens [20,23,24,25]. The properties of these probiotic strains have made them promising poultry feed additives. Among yeast, *Saccharomyces* are effective in the degradation of dietary phytate to augment the quality of food [26,27]. *Saccharomyces* cell wall components, beta-glucan, and mannanoligosaccharides, are accountable for their favorable impacts on performance [24,28,29,30] via promoting the gut microbiota and histology, decreasing the pathogenic bacteria count, and improving digestive enzyme activities [24,29,30]. Akhavan-Salamat et al. [31] reported that *Saccharomyces cerevisiae* addition at a level of 0.2% to a reduced P diet augmented the appetite and performance, nutrient digestibility, and the availability of P and Ca for broilers.

To date, there is little research on the effect of multi-strain probiotics, *Saccharomyces cerevisiae*, and fumaric acid on the performance, P utilization, bone quality traits, and intestinal P transporter genes in broiler chickens fed a reduced P diet. Our hypothesis was that the addition of a multi-strain probiotic, *Saccharomyces cerevisiae,* or fumaric acid to a low nPP diet could be useful in decreasing the adverse effects of low dietary nPP in broiler chickens and might be comparable to phytase addition. Thus, the objective of the current trial was to determine the efficacy of phytase, multi-strain probiotic, *Saccharomyces cerevisiae*, and fumaric acid supplementation to a reduced nPP diet on performance, carcass traits, nutrient digestibility, bone physical traits and mineralization, tibia and intestinal histomorphology, blood constituents and hormone profile, and intestinal phosphorus transporter genes of broiler chickens.

## 2. Materials and Methods

### 2.1. Ethical Approval

The provision and procedure applied for birds in this study were approved by the Institutional Animal Care and Use Committee (IACUC), Faculty of Veterinary Medicine, Sadat City University (Ethical approval number: VUSC-016-1-21).

### 2.2. Feed Additives

The phytase enzyme used in the present trial was Natuphos^®^ (10,000 FTU/g) and was provided by BASF (Ludwigshafen, Germany). The multi-strain probiotic used in the present trial was supplied by the Microbiological Resources Center (MIRCEN; Cairo, Egypt). The probiotic consisted of *Lactobacillus acidophilus* 2 × 10^10^ CFU/g, *Lactobacillus plantarum* 2 × 10^10^ CFU/g, *Enterococcus faecium* 1 × 10^9^ CFU/g, *Bacillus subtilis* 2.1 × 10^8^ CFU/g, and *Aspergillus awamori* 2.5 × 10^4^ CFU/g. Dried active yeast (*Saccharomyces cerevisiae*) was supplied by The Egyptian Company for Starch, Yeast, and Detergents, Co. (Alexandria, Egypt) and contained 3.14 × 10^8^ CFU *Saccharomyces cerevisiae*/g. The fumaric acid (99.9%) was supplied by Egypt Veterinary Medicines and Feed Additives, Co. (EVPCO, Alexandria, Egypt).

### 2.3. Experimental Design

A total of 480 one-day-old Arbor Acres plus broiler chickens were supplied by a local hatchery (Arab for Poultry Breeders Co., Ltd., Giza, Egypt). On arrival, broiler chicks were weighed and randomly assigned to six groups of eight replicates (5 ♂ + 5 ♀/replicate/pen; *n* = 80 per group) following a completely randomized design. The trial continued for 5 weeks (from day 1 to d 35 of age). The raising period was divided into 3 phases, 1–10 days (starting), 11–24 days (growing), 25–35 days (finishing) of age. The broilers were handled according to the guidelines of the breed (Arbor Acres Broiler Commercial Management Guide). During the experimental period, broilers were raised on floor pens in a well-organized environment. The starting temperature was 32 °C during the first seven days and then gradually decreased to 24 °C and maintained at 24 °C till the end of the experiment. A lighting schedule was provided for 24 h during the first 3 days and then sustained for 23 h light and 1 h darkness. The vaccination program was performed in accordance with the breeder standards.

Broilers were fed one of six dietary treatments: (1) a positive control (PC), a diet containing the recommended Ca and nPP levels (nPP; 0.48, 44, and 41% in the starting, growing, and finishing periods, respectively); (2) a negative control (NC), a diet contained the recommended Ca level and reduced nPP (0.28, 24, and 21% in the starting, growing, and finishing periods, respectively); (3) NC + 600 FTU/kg phytase (PHY); (4) the NC diet + 0.05% multi-strain probiotic (PRO); (5) NC + 0.2% *Saccharomyces cerevisiae* (SC); and (6) NC + 0.2% fumaric acid (FA). Feed constituents and proximate chemical analyses of the experimental diet are shown in Table 1. Chromic oxide was included in the investigational diets as an indigestible marker (3 g/kg of diet) in the finishing dietary period of the experiment for evaluating the digestibility of nutrients. Broiler chickens had ad libitum access to feed and water during this study. Dietary samples were collected and analyzed for proximate chemical composition following AOAC [32] (Table 1). Dietary phytate P was determined [33], and available P was calculated as total P minus phytate P.

### 2.4. Production Performance and Sampling

Body weight (BW) and feed intake (FI) for each replicate were documented weekly, and consequently, BW gain (BWG) and feed conversion ratio (FCR, g feed/g gain) were estimated. Mortality was reported on a daily basis in each experimental group. At 21 and 35 days of age, a total of 96 broilers (2 birds (1 ♂ + 1♀) from each pen; 16 broilers per group), within the average BW of the group, were chosen for blood chemical analysis. Blood samples were obtained from bird wing veins and centrifuged (at 3000 rpm for 15 min) to obtain the serum. Serum samples were stored at −20 °C till further blood chemical analyses. Additionally, at 35 days of age, a total of 96 broilers (2 birds (1 ♂ + 1 ♀) from each replicate/pen), within the average BW of the group, were selected for carcass parameters, bone analyses, gut histology, and gene expression analysis. Blood samples were taken from the bird’s wing vein, centrifuged (at 3000 rpm for 15 min) to obtain serum, and kept at −20 °C for additional chemical analyses. Then, birds were euthanized by cervical dislocation. The tibia was gathered from each chicken to estimate bone mineralization. The tibia bones were cleaned from all attached tissue and kept frozen at −20 °C. A 2 cm piece of the midpoint of the small intestine was removed. In addition, duodenal tissue samples were collected and maintained frozen at −80 °C to evaluate P transporter gene expression. The weights of the carcass, liver, heart, gizzard, and lymphoid organs were recorded and represented as % of BW.

### 2.5. Sample Collection

To evaluate the digestibility of nutrients, three days before the end of the experiment, chromic oxide was included in the experimental diets at the expense of maize. Excreta samples were gathered at trial end with caution to prevent contamination from foreign materials and immediately kept at −20 °C for further analyses. The diet and excreta samples were finely ground and thoroughly mixed for proximate chemical composition. The dry matter (DM), crude protein (CP), ether extract (EE), ash, calcium (Ca), and P were evaluated in the samples according to AOAC [32]. The amount of chromic oxide in the diet and excreta was measured as described by Lomer et al. [34].

Nutrient digestibility (%) = 100 − [(TiO_2_ diet/TiO_2_ excreta) × (total nutrient excreta/total nutrient diet) × 100].

### 2.6. Serum Parameters

The serum constituents, including total cholesterol (TC), triglycerides (TG), glucose, Ca, P, and alkaline phosphatase (ALP), were estimated spectrophotometrically (ultraviolet spectrophotometer UV4802, Unico Co., Dayton, OH, USA) with available kits (Bio-diagnostic Co., Cairo, Egypt) following the manufacturer’s recommendations. Serum triiodothyronine (T3) and thyroxine (T4) concentrations were determined by ELISA with an available ELISA kit (IBL International GmbH, Hamburg, Germany).

### 2.7. Parameters of Bone Quality

The left tibia was removed and cleaned from all adherent tissues, including cartilage caps, and then stored at −20 °C until bone mineral analyses. Tibia weight (g) was measured by a decimal digital scale, tibia length and width (mm) were determined using a digital micrometer. Tibia breaking strength was assessed and represented in kilograms’ force required to break the bone following the procedure of Flemming et al. [35]. The left tibia bones were crushed and dried for 24 h at 105 °C in a hot air oven. The bones were defatted by a Soxhlet apparatus with petroleum ether for 24 h and then dried at 100 °C for 24 h. The dried fat-free tibia was muffled in a muffle furnace at 600 °C overnight, and tibia ash content was expressed as a percentage of fat-free dry basis. Tibial ash, Ca, and P levels were measured by the same methods as those used for feed and excreta samples [32].

### 2.8. RNA Extraction and Quantitative Real-Time PCR

Total RNA was extracted from duodenal mucosal samples with TRIzol isolation kits (Life Technologies Ltd., Renfrew, UK) following the manufacturer’s recommendations. Purity and concentration of the total RNA were checked by NanoDrop™ 2000/2000c Spectrophotometer (Thermo Fisher Scientific, Waltham, MA, USA) at 260 and 280 nm (A260/280 ratio), and the purity of extracted RNA was 1.8 to 2. The integrity was checked by 1% agarose gel electrophoresis. cDNA was reverse transcribed using TOPscript™ RT DryMIX (dT18/dN6) according to the manufacturer’s direction (catalog no. RT220, enzynomics, Inc., Korea). Quantitative real-time RT-PCR was performed in triplicates in the thermal cycler (AriaMx Real-time PCR System) using the TOPreal™ SYBR Green qPCR PreMIX (catalog no. RT5005, Enzynomics, Inc., Daejeon, Korea). The amplification reaction of PCR was performed as follows; initial denaturation at 95 °C for 10 min, followed by 45 cycles of 10 s at 94 °C, 60 °C for 35 s, and 70 °C for 35 s. The primers of the internal control gene and target genes were previously described by Hu et al. [36]. The internal control gene was β-actin, and the studied genes were type IIb sodium-dependent phosphate co-transporter (NaPi-IIb), inorganic phosphate transporter 1 (PiT-1), and inorganic phosphate transporter 2 (PiT-2). Relative mRNA expression abundance of the considered genes was estimated using the 2^−ΔΔCT^ method [37].

### 2.9. Gut and Tibia Histomorphology

Intestinal segments (duodenum, jejunum, and ileum) of approximately 2 cm were removed [38] and fixed in 10% buffered formalin for seven days and used to determine villus height (VH) and width (VW), crypt depth (CD), and muscular thickness (MT). Tissues of the small intestine were dehydrated by dipping in alcohols of sequentially elevated concentrations (from 70 to 100%), infiltrated with xylene, and inserted in paraffin. Sections were then stained with hematoxylin and eosin for measuring the morphometric parameters. These parameters were determined by randomly assessing 10 villi and expressed as micrometers (µm) [38,39] using ImageJ software. Furthermore, villus absorptive surface area was estimated by the subsequent equation [29]:

Villus absorptive surface area = 2π × (average VW/2) × VH

Tibia bone samples from the right leg were fixed in formalin for approximately 48 h and then transferred to a decalcified solution, which is necessary to permit effective tissue sectioning by microtome but to overcome over-decalcification to keep cellular contents intact [40,41]. The typical solutions applied for decalcification are hydrochloric acid, formic acid, and ethylene diamine tetra acetic acid. In the current study, we used 8% formic acid for about two weeks till decalcification occurred to osseous tissue. After that, the decalcified bones were washed under tap water several times and dehydrated by ascending grades of alcohols (70 to 100%). Then, they went through a clearing agent by xylol and mounting by paraffin. Tissue blocks were sectioned by a rotatory microtome at 4–6 µm, mounted on clean glass slides, and stained by hematoxylin and eosin stain. Tibia histomorphological parameters were performed on trabecular, cortical, and medullary regions using ImageJ software, following the methods of Pérez Castrillón et al. [42]. The following parameters were measured: cortical thickness (µm), medulla thickness (µm), trabecular thickness (µm), trabecular number (1/mm^2^), and trabecular separation (µm).

### 2.10. Statistical Analysis

The normality of the data distribution was assessed with the Kolmogorov–Smirnov test before statistics. All data were exposed to One-way ANOVA as a completely randomized design using the IBM SPSS statistical package (version 22, SPSS Inc., Chicago, IL, USA) to determine the influence of treatments, together with Duncan’s multiple-range test (*p* < 0.05). The experimental unit for evaluating growth performance traits was the pen and the bird for other traits. Variations between NC and other groups were compared by orthogonal probability contrasts.

## 3. Results

### 3.1. Growth Performance

The growth performance data of the experimental groups are shown in Table 2 and Table 3. There was non-difference in the initial BW between the treatment groups. All treatment groups, except the FA group, improved BW (*p* < 0.01) from week 3 to week 5 compared to NC (Table 2). During the first two weeks of age, the PHY and PRO broilers had greater BW (*p* < 0.01) than the NC and PC broilers (Table 2). The final BW and total BWG in the whole trial period (1–35 d) of broilers fed with the PHY (*p* < 0.001), PRO (*p* < 0.001), and SC (*p* < 0.01) diets were better than those fed the NC diet, into which those fed the PRO and PHY diets were the greatest. Nevertheless, there were non-differences in the final BW and total BWG between the NC and FA broilers. At 7 days of age, all treatment groups were shown to have a decreased daily feed intake (*p* < 0.001) in comparison with the NC group. At week 5, only the PRO broiler chickens had a lower average daily FI (*p* < 0.01) than the NC ones. Generally, during the trial, a non-significant difference in the cumulative FI among treatments was observed (Table 3). Our findings of the whole experimental period (d 1 to 35) observed that the PHY (*p* < 0.05) and PRO (*p* < 0.01) treatments had improved FCR than NC, while PC, SC, and FA treatments were not significantly varied from NC (Table 3). The performance of broilers fed the FA diets did not differ from that of NC.

### 3.2. Carcass Traits and Relative Organs’ Weights

Table 4 summarizes the effect of supplemental PHY, PRO, SC, and FA to low nPP diets on broilers’ carcass characteristics at 35 days of age. There was a non-significant impact of the various dietary treatments on the relative heart, gizzard, and thymus gland weights as compared to NC. However, the supplementation of PHY and PRO (*p* < 0.01) to a low nPP diet improved the dressing % compared to NC, whereas the dressing % of the PC, SC, and FA treatments were not varied from that of NC. Greater relative liver weight was observed in FA compared to NC (*p* < 0.01), whereas there were non-variations in the liver percentage between PC, PHY, PRO, or NC. Broilers fed the PHY, PRO, SC, and FA diets had higher (*p* < 0.001) relative weights of spleen and bursa of Fabricius (within the normal values) than those fed the low nPP diet.

### 3.3. Nutrient Digestibility

The digestibility of DM (*p* < 0.001) and P (*p* < 0.001) was reduced in broilers fed the low nPP diets than those in other treatments (Table 5). The Ca and EE digestibility were greater (*p* < 0.01) in the PHY, PRO, and SC groups than in the NC group, although there was a non-difference in the Ca and EE digestibility between PC, NC, and FA. Additionally, the digestibility of CF in chickens fed the low nPP-supplemented diets was improved (*p* < 0.01) compared to those fed the low nPP diet. The CP digestibility was improved (*p* < 0.001) in broilers fed a diet with reduced nPP and supplemented with PHY, PRO, SC, and FA as compared to the low nPP and PC diets.

### 3.4. Blood Biochemical and Hormones Profile

Table 6 showed the impact of dietary treatments on serum constituents and hormone profile of broilers at 21 days of age. Serum concentrations of Ca (*p* < 0.01) and P (*p* < 0.01), and T4 (*p* < 0.001) were higher in broilers fed with the PC and all supplemented diets than those fed the low nPP diet. On the contrary, decreased serum levels of TG and glucose were observed (*p* < 0.01) in the low nPP-supplemented groups compared with the low nPP and PC groups. There was a non-significant variance in the serum concentrations of TG and glucose between PC and NC treatments. The TC concentration in serum was lower (*p* < 0.001) in the NC and NC-supplemented broilers compared to the PC ones. In addition, its concentration was lower (*p* < 0.001) in PHY, SC, and FA than in NC. Serum T3 concentration was increased (*p* < 0.01) in birds fed with the PHY, PRO, and FA diets than in those fed with the NC diet, whereas there was non-significant variation in T3 between NC, PC, and SC. The serum concentration of ALP was not different among treatments.

The data in Table 7 summarize the effect of various supplements to a low nPP diet on serum constituents and hormones of broiler chickens at 35 days of age. Serum Ca and P levels were greater (*p* < 0.001) in broilers fed the PC and low nPP-supplemented diets than those fed with the low nPP diet. Conversely, elevated serum TG and TC levels were observed (*p* < 0.001) in the low nPP group compared to other treatments. There was non-significant variance in the serum levels of glucose between the PC, NC, SC, and FA groups; whereas, the PHY and PRO groups had lower serum glucose concentrations in comparison to NC. Lower serum T3 and T4 concentrations (*p* < 0.001) were found in broilers fed with NC diets compared to other treatments. The serum level of ALP was higher (*p* < 0.001) in the NC group when compared with the PC and NC-supplemented groups.

### 3.5. Bone Characteristics

Physical and chemical tibia characteristics of broiler chickens fed with the different dietary treatments at day 35 of age are shown in Table 8. Tibia weight, length, and width were increased (*p* < 0.001) when PHY, PRO, SC, and FA were supplemented with the low nPP diets. By comparison to the low nPP group, the greatest values (*p* ˂ 0.001) for tibia weight and length were observed for broiler chickens fed the PHY and PRO diets, whereas the greatest tibia width (*p* < 0.001) was shown in the broiler chickens fed with the SC and FA diets. Regarding the breaking strength of the tibia, the PC, PHY, PRO, SC, and FA treatments led to greater tibia breaking strength (*p* < 0.001) than did the low nPP group, where the greatest values were noted for the PRO group. Decreasing dietary nPP level reduced (*p* < 0.05) tibia weight, length, width, and breaking strength compared with PC. Tibia ash, Ca, and P concentrations in broiler chickens fed with the PHY, PRO, SC, and FA were greater (*p* < 0.001) than that in broilers fed with the NC diets. By comparison to PC, reducing the nPP level in the broiler chickens’ diets resulted in a reduction (*p* < 0.001) in the ash and P contents, whereas there was a non-significant difference in the tibia Ca content between PC and NC. Tibia DM and Ca:P were not different among treatments.

### 3.6. Gut Histology

As shown in Table 9, reducing nPP levels in the diet of broiler chickens (NC) decreased duodenal VH, VW, MT, VH:CD, and VSA (*p* < 0.01) when compared with those fed on the PC and NC-supplemented diets. In the jejunum, VH, VW, MT, CD, and VSA were increased (*p* < 0.01) in the PC, PHY, PRO, SC, and FA broilers as compared to the NC ones. In comparison to NC, reducing dietary nPP levels with PHY, PRO, SC, and FA supplementation in the broiler chickens resulted in an increase in the ileal VH and VW, CD, MT, and VSA (*p* < 0.01).

### 3.7. Bone Histomorphology

**Figure 1:**
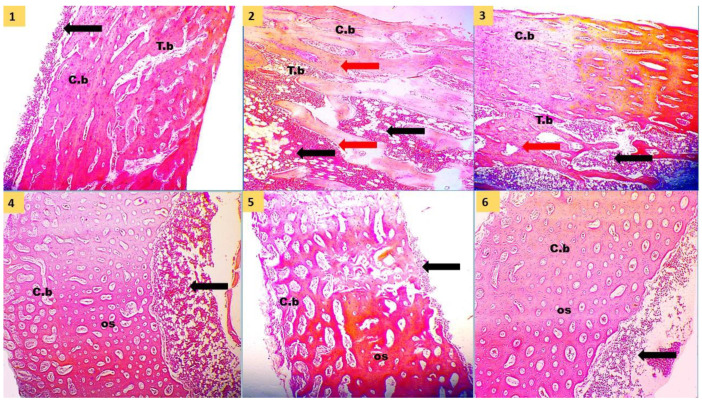
Photomicrograph of the histological structure of diaphysis of tibia in broiler chickens at 35 d of age showed cortical compact bone (C.b) with its osteons (os), medullary trabecular bone (T.B) with its bone trabeculae (red arrow), and bone marrow (black arrow). Hematoxylin and eosin stain (H & E, x40). (**1**), Positive control diet contained the recommended Ca and nPP; (**2**), negative control diet contained the recommended level of Ca and low nPP; (**3**), NC + 600 U phytase/kg of the diet; (**4**), NC + 0.05% multi-strain probiotic; (**5**), NC + 0.2% *Saccharomyces cerevisiae*; and (**6**), NC + 0.2% fumaric acid.

**Figure 2:**
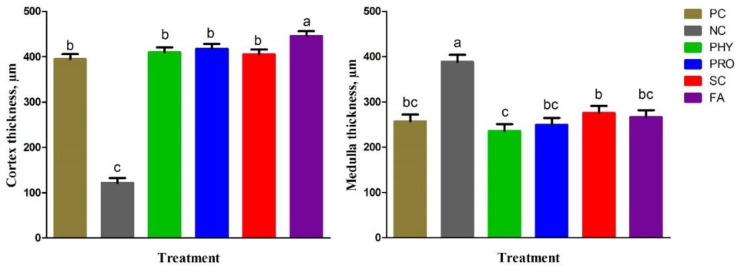
Effect of dietary treatments on cortical bone thickness and medullary area of tibia in broiler chickens at 35 d of age. Data represented as mean ± SEM. ^a–c^ Means with different letters varied at *p* ˂ 0.05. For treatment abbreviations, see Table 2.

**Figure 3:**
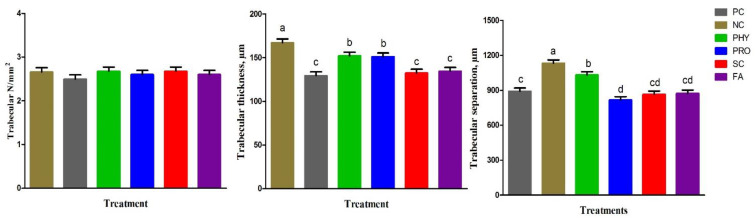
Effect of dietary treatments on trabecular bone properties of tibia in broiler chickens at 35 d of age. Data represented as mean ± SEM. ^a–c^ Means with different letters are varied at *p* ˂ 0.05. For treatment abbreviations, see Table 2.

Figure 1 shows the tibia histological structure of the experimental groups at day 35 of age. The histological appearance of the diaphysis of the tibia bone showed normal bone structure formed from cortical compact bone and medullary cancellous or trabecular bone in the PC group. In particular, the PC, PHY, PRO, SC, and FA groups showed normal and thicker compact bone structures where bone lamellae organized to form Haversian lamellae (osteones), interstitial lamellae, and outer and inner circumferential lamellae. Whereas the NC group represented a thicker medullary cancellous bone, appearing as the bone lamellae forming branching and anastomosing trabeculae and plates, and between the trabeculae, there were wide spaces filled with bone marrow. Moreover, the medullary bone was free from bone marrow in the other groups. Figure 2 and Figure 3 represented the histomorphological measurements of the tibia in broiler chickens fed the dietary treatments at day 35 of age. The NC group showed thinner cortical bone thickness (*p* < 0.001) and thicker medullary bone thickness (*p* < 0.01) than PC, while the supplemented groups had thinner medulla (*p* < 0.01) and thicker bone cortex (*p* < 0.001) when compared with the NC group. The FA group recorded the highest cortical tibia thickness compared to other treatments. Compared to the low nPP group, all nPP-supplemented groups had thinner medulla thickness (*p* < 0.01) and were comparable to the PC group. The thickness of trabeculae was reduced (*p* < 0.001), while the trabecular separation enlarged (*p* < 0.01) in the NC group compared to the PC group. The trabecular thickness in the PHY and PRO was increased when compared with that of the NC group. The number of trabeculae of the tibia was not different among the treatment groups.

### 3.8. Intestinal Gene Expression

**Figure 4:**
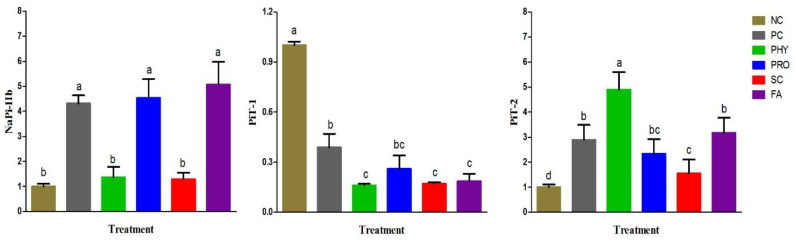
Effect of dietary treatments on phosphorus transporter gene expression of broiler chickens at 35 d of age. Expression of NC is taken as 1.0. Data represented as mean ± SE. ^a–c^ Means with different letters varied at *p* ˂ 0.05. For treatment abbreviations, see Table 2. NaPi-IIb, type IIb sodium-dependent phosphate co-transporter; PiT-1, inorganic phosphate transporter 1; PiT-2, inorganic phosphate transporter 2.

The results of duodenal NaP-IIb, PiT-1, and PiT-2 mRNA levels at 35 d of age are presented in Figure 4. The expression level of NaP-IIb increased (*p* ˂ 0.001) in broilers fed the PRO and FA diets compared to the NC, PHY, and SC ones and had a similar magnitude to that of PC. Moreover, there was a non-significant variation in NaP-IIb expression between the NC, PHY, and SC groups. Birds fed the low nPP diet showed greater expression of PiT-1 (*p* ˂ 0.01) than other treatments. By comparison to NC, all nPP-supplemented groups showed up-regulation (*p* ˂ 0.01) of PiT-2 mRNA expression level.

## 4. Discussion

In the corn-SBM-based diets, approximately 60% of P is bound to phytate and unavailable to broiler chickens. The most effective approach to increase P availability and utilization is via dietary supplementation with phytase, feed additives with phytase-like activity, or by making gut pH more favorable for phytase to hydrolyze phytate. Therefore, the main objectives of the present trial were to investigate if the supplementation of PHY, multi-strain PRO, SC, or FA to a reduced nPP diet could have beneficial impacts on performance, nutrient digestibility, bone mineralization, bone and gut histomorphology, blood constituents and hormone profile, and duodenal P transporter genes of broiler chickens.

The findings of the current study show that BW and BWG of broilers fed the PHY, PRO, and SC diets were better than those fed the low nPP and FA diets, where those fed PRO and PHY diets had the greatest BW and BWG and were comparable to that of the PC broilers. Moreover, overall, there were non-significant variations in FI among the treatment groups. The PHY and PRO groups had better FCR than the PC group, and the FA and SC groups had FCR comparable to that of the PC group. Noticeably, these supplements could replace a portion of nPP (0.2%) in the diet of broiler chickens without inducing any negative effect on performance. The reduction of growth performance and FCR due to low dietary nPP levels confirmed the incapability of these birds to utilize phytate P. Furthermore, the recommended nPP levels (without phytase) were sufficient to maintain growth performance, and in agreement with previous studies [7,10,43,44]. The enhancement in the growth rate of broilers fed low nPP diets supplemented with PHY can be mainly contributed to increasing P availability, alleviating the negative impacts of phytate on various nutrients’ digestibility, and enhancing energy utilization [7]. The beneficial effect of dietary PHY on the growth performance of broilers reported in this study was in agreement with earlier studies [7,10,43,44].

The multi-strain PRO used in the current study was a combination of microorganisms that were reported to have phytase-like activity and are able to facilitate the absorption and utilization of P and decrease P excretion, such as *Lactobacillus* spp. [17,18,19], *Enterococcus faecium* [20], *Bacillus subtilis* [19,21,45], and *Aspergillus awamori* [22]. These microorganisms, either in combination or individually, were reported to augment the growth rate of broilers via maintaining a good intestinal microbial balance which, in turn, augments P availability, boosts gut integrity and immunity, and avoids enteric pathogens [20,23,24,29]. Therefore, the observed improvement in the growth performance of broilers fed PRO can be attributed to the above-mentioned impacts. Besides having probiotic effects, *Saccharomyces* were shown to be efficient in the degradation of phytate to augment food value [26,27] and SC cell wall components, for example, beta-glucan and mannanoligosaccharides. Furthermore, they can be accountable for beneficial effects on growth performance [24,28,29,30] via improving the gut microbiota and decreasing the growth of pathogenic bacteria, which, in turn, increase intestinal enzyme activities and nutrient digestion and absorption, resulting in better growth performance [24,29,30]. Our findings are consistent with Akhavan-Salamat et al. [31], who showed that SC addition at a level of 0.2% to a reduced P diet augmented the appetite and growth performance of broiler chickens. In the current study, the BW and BWG of broiler chickens fed with FA diets were numerically greater than those of the NC group but lower than those of the PC group. There was a non-significant difference in FI or FCR between FA, NC, or PC. On the contrary, Nari and Ghasemi [10] observed that dietary 0.2% butyric acid supplementation to low nPP diets (0.1% reduced in nPP) improved BW with no difference in FI or FCR. Our results are consistent with Liem et al. [13], who found that the addition of FA to a low P diet did not increase BW or gain-to-feed ratio.

The observed enhancement in feed efficiency of low nPP-supplemented groups, particularly PHY, PRO, and SC, in the current trial can be elucidated by greater nutrient digestibility (DM, CP, EE, P, and Ca). The positive effects of PHY on nutrients’ digestibility may contribute to the ability of PHY to destruct phytate, the availability of nutrients beyond P, the decrease of intestinal losses of amino acids and minerals, and enhanced the activity of endogenous enzymes [7,44]. These findings are inconsistent with previous research [8,10,44]. The phytase-like activities of multi-strain PRO induced beneficial digestive impacts by enhancing the digestion and utilization of nutrients which are typically accomplished by the breakdown of phytic acid in the digestive tract. Furthermore, the positive effect of PRO on nutrient digestibility could be related to the synergistic effect of the selected probiotic microorganisms that can adjust the intestinal microbiota, decrease digestive disorders, prevent the growth of pathogenic microorganisms [20,23], lower the intestinal pH, which can help in the digestion and absorption of protein and minerals [25], and increase the intestinal and pancreatic enzymes [46]. Regarding SC, Akhavan-Salamat et al. [31] observed that the supplementation of SC to a low P diet enhanced the CP, P, and Ca ileal digestibility, and they stated that the ability of SC to encourage nutrient digestion and retention might be partly associated with the phytase-like activity of SC, which enabled better utilization of phytate P [31]. A decline in the gastric pH as a result of organic acid addition led to a better conversion rate of pepsinogen to pepsin, encouraged pepsin activity [46], and decreased the synthesis of mineral–phytate complexes [15], therefore augmenting the digestion and utilization of protein and minerals [10,15]. Our findings are in agreement with Nari and Ghsemi [10], who reported that butyric acid and *Saccharomyces boulardii* supplementation to a low P diet improved CP, Ca, and P digestibility. Additionally, Sileikiene et al. [46] recorded greater trypsin and amylase activities and pancreatic fluid secretion by dietary butyric acid addition. Improving nutrient digestion and utilization through dietary PRO, SC, and FA supplementation offers a practical and cost-effective strategy to decrease excessive P excretion into the environment and consequently lessening its involvement in environmental pollution.

The supplementation of PHY and PRO to a low nPP diet increased the dressing % compared to the NC group and even greater than those of the PC group. Consistent with the current results, El-Faham et al. [47] observed that reducing Ca and nPP in the diet of broiler chickens to 50% of the recommendations reduced the dressing and carcass yields. Moreover, Han et al. [48] recorded that a lower Ca to nPP ratio caused a decrease in the carcass yield of broiler chickens. In contrast, Imari et al. [49] recorded that limiting Ca and nPP levels by 10% to 30% of the requirements in broilers’ diets did not significantly influence the carcass parameters and internal organs. In this study, the observed higher spleen and bursa of Fabricius percentages (within the normal values) in the PRO, SC, and FA broilers suggest a better immune response in these groups and support the beneficial effects of the studied feed additives on broilers’ health. Greater indexes of immune organs are generally estimated as indicators of the enhanced proliferation of B- and T-lymphocytes, which indicates improved immunity [50,51]. Nari et al. [52] observed that broilers fed a reduced nPP diet (by 0.1%) supplemented with butyric acid and *Saccharomyces boulardii* had greater immune organ weights and antibody response. The increase of the immune organ weights may also be associated with the positive impact of these supplements on the gastrointestinal microbiota [50,51], into which the increase in gastrointestinal *Lactobacillus* spp. was associated with the effectiveness of the host’s immunity [53]. However, in the current study, we did not analyze gut microbiota to confirm this speculation, and thus it warrants further research.

The thyroid gland is accountable for the secretion of T3 and T4, which play important roles in controlling various metabolic pathways and nutrient metabolism, involving Ca and P homeostasis. In the current study, feeding a low nPP diet resulted in a reduction in the serum level of T3 and T4 compared with the PC diets, suggesting that low dietary available P intake can negatively influence thyroid hormone functions [10]. Thus, the augmented capacity to utilize phytate P could reveal the greater T3 and T4 levels in the serum of the NC-supplemented groups. Since T3 and T4 hormones have an effect on growth rate through numerous metabolic pathways [54], elevated serum levels of thyroid hormones in the NC-supplemented groups can clarify its better BWG [10]. Reduced P levels in the serum of broiler chickens fed with the NC diet were in line with the previous studies [10,13,44]. Birds fed the PHY, PRO, SC, and FA diets had greater serum P and lower ALP activity than NC, indicating higher P availability than NC due to the action of these supplements on phytate-bound P [10,44]. Elevated blood ALP activity is known to be related to bone disorders [12] and might be linked to Ca or P-insufficiency or a higher Ca to P ratio in the diet. The addition of PHY [10,44], organic acids [10,13], or *Saccharomyces* [10,31] to low P diets reduced blood ALP activity, possibly as a result of the down-regulation of this enzyme because of the augmented accessibility of P [55]. In this investigation, decreases in serum ALP activity may suggest that the P level can regulate ALP production.

In the present study, the addition of dietary supplements to a low nPP diet decreased serum TC and TG concentrations when compared with the NC and PC groups. These findings were contrary to previous findings, which reported no effect of dietary PHY supplementation to low P diet [44] or low Ca and P diet [56] on blood TC and TG concentrations. Little information is available in the literature about the effect of the studied supplements with low nPP diet on blood lipid profile to compare with the results reported herein. Our findings were consistent with Li et al. [57], who observed that broiler chickens fed an adequate P diet had greater serum TC and TG levels than those fed a P-deficient diet, and they suggested that this may be due to TG being used to supply energy for life activities [57]. Hussein and Selim [24] and Saleh et al. [58] reported that *Aspergillus* and *Saccharomyces* supplementation reduced blood TC and TG concentrations in chickens. The mechanism describing the impact of PRO and SC on reducing serum TC can be contributed to the suppression of 3-hydroxyl-3-methylglutaryl-coenzyme reductase and deconjugation of intestinal bile salts, therefore diminishing cholesterol synthesis [24,58]. The capability of dietary FA supplementation to decrease microbial intracellular pH may clarify the significant decrease in serum TC in the FA broilers [59,60], and our results were in line with those of Reda et al. [59] and Kamal and Ragaa [60].

Tibia physical traits and mineralization were improved by PRO, SC, and FA addition to the low nPP diet and were comparable to the PC and PHY groups. P is important for bone development and various tissues in the rapid-growing birds. It was reported that supplementation of low-nPP diets with 0.2% organic acid mixture [15] or 0.2% butyric acid [10] improved tibia breaking strength and ash content. The improvement in tibia breaking strength and mineralization in the supplemented groups can be due to the enhancement in phytate P availability. Organic acids found in the dissociated form were effective in enhancing phytate P availability, primarily due to their capacity to decrease the gut pH for a better environment for phytate breakdown [13,14]. Regarding PRO, the improvement in tibia ash, Ca, and P contents might be due to the phytase-like activities and lowering of the intestinal pH by a multi-strain PRO, which achieved better conditions for degradation of phytate P [25]; however, the phytase activity and gut pH were not measured in the current study to confirm our findings. The ability of SC to encourage tibia ash and P concentrations could be related to the phytase-like activity of SC, which enabled better utilization of phytate P [31]. Suzer et al. [61] observed that SC supplementation at a level of 0.2% resulted in higher tibia ash content and greater breaking strength than the control diet. Improved tibia breaking strength and mineralization in the supplemented groups were associated with greater cortical area thickness. Williams et al. [62] recorded that rapid-growing birds had a greater cortical thickness than slow-growing ones. In the current trial, low nPP broilers had greater medullary thickness while cortical thickness decreased, which may indicate resorption of endosteum via enhanced osteoclastic activity at the endosteal surface and a decrease in the periosteal osteoblastic activity for new bone formation [62,63]. Our results suggested that supplementary PRO, SC, and FA to the low available P diet can positively maintain tibia mineralization, histological structure, and development to the same levels as the adequate nPP diet as a result of better P absorption, utilization, and deposition.

In the current trial, dietary PHY, PRO, SC, and FA supplementation to a reduced nPP diet resulted in greater VH and VW, CD, MT, and VSA compared to the low nPP-diet, and their values were comparable to the PC ones. VH and VW, CD, MT, and VSA of the small intestine are all crucial factors for determining intestinal health, nutrient digestion, absorption, and assimilation [64,65]. Shortening the height of villi or decreasing villi CD may lead to a diminished capacity of the intestine to absorb nutrients [64,65]. Our findings are consistent with previous studies that recorded a greater VH of small intestinal epithelium in broiler chickens fed diets supplemented with PHY [52,66], probiotic [29,67], SC [68], and organic acids [66,67]. Recently, Nari et al. [52] observed that dietary supplementation of PHY, *Saccharomyces boulardii*, and butyric acid to a low-nPP diet improved duodenal and jejunal VH and VH:CD compared to the NC diet. Owing to the fact that gut health is necessary to the growth performance and health status of broiler chickens [55,64], the enhancement in the performance and nutrient digestibility in the NC-supplemented groups can also be clarified by improved intestinal histomorphology and well-adjusted gut microbiota [52].

NaP-IIb is predominantly expressed in the duodenum and is known as an essential P transporter in the small intestine of broiler chickens [36]. Hu et al. [36] reported that PiT-1 and PiT-2 might play a part in the regulation of P absorption in broiler chickens. In our study, the duodenal mRNA expression of NaP-IIb was up-regulated in the PRO and FA groups, which may be owing to an improvement in the intestinal P availability and absorption since its expression level increases as dietary P increases, and is concentration-dependent [20,36]. Low gastric pH induced by organic acids and phytase-like activities of PRO can facilitate the enhancement of phytase activity by gut microbiota which in turn up-regulated NaP-IIb. Increasing dietary nPP stimulated PiT-2 mRNA and protein expressions and diminished PiT-1 mRNA and protein expression [36]. Therefore, the up-regulation of the duodenal PiT-2 mRNA in all nPP-supplemented groups may suggest increased intestinal P availability in these groups. Although, the major role of PiT-1 and PiT-2 in the transcellular P absorption and the post-transcriptional regulation of both transporters in the case of low nPP-supplemented diets need to be further investigated in broiler chickens.

## 5. Conclusions

The dietary addition of PHY or PRO to the low nPP diet was beneficial compared with SC and FA with respect to performance and carcass traits. Supplementary PRO, SC, and FA to a reduced nPP diet enhanced intestinal P absorption and utilization and positively maintained tibia structure to the same levels as the adequate nPP diet. Therefore, it can be concluded that these dietary supplements to low nPP diets were beneficial in mitigating the detrimental effects of reduced dietary P in broiler chickens. Further investigation is needed to determine the effect of these supplements on the gut microbiota and the intestinal protein levels of P transporter genes.

## Figures and Tables

**Table 1:** Composition and analysis of the experimental diets.

Items	Experimental Diets ^1^					
	Starter 1–10 d		Grower 11–24 d		Finisher 24–35 d	
	PC	NC	PC	NC	PC	NC
Ingredients, %						
Yellow Corn	52.93	53.26	57.22	57.51	58.93	59.00
SBM, 44% CP	36.10	36.41	32.40	32.40	30.95	31.17
Corn gluten, 62%	4.00	4.00	4.00	4.00	2.50	2.50
Vegetable oil	3.00	3.00	3.10	3.10	4.60	4.60
Dicalcium phosphate	1.78	0.73	1.62	0.55	1.47	0.413
Limestone	1.26	1.70	0.83	1.62	0.79	1.57
dl-methionine	0.156	0.152	0.124	0.123	0.122	0.120
l-lysine	0.121	0.115	0.083	0.083	0.04	0.03
l-threonine	0.05	0.04	0.02	0.014	-	-
Common salt	0.30	0.30	0.30	0.30	0.30	0.30
Premix ^2^	0.30	0.30	0.30	0.30	0.30	0.30
Total	100	100	100	100	100	100
**Chemical composition**						
ME, MJ/kg	12.55	12.57	12.96	13.00	13.39	13.40
Crude protein, %	22.96	23.03	21.50	21.60	20.00	20.07
Calcium, %	0.96	0.96	0.87	0.87	0.81	0.81
Available Phosphorus, %	0.48	0.28	0.44	0.24	0.41	0.21
Total Lysine, %	1.44	1.44	1.29	1.29	1.19	1.19
Total Methionine, %	0.56	0.56	0.51	0.51	0.48	0.48
Total Threonine, %	0.97	0.97	0.88	0.88	0.81	0.81
**Analyzed composition**						
Dry matter %	90.5	90.4	89.80	89.76	89.7	90.1
Crude protein, %	22.89	22.96	21.47	21.52	19.93	20.00
Calcium, %	0.96	0.96	0.88	0.86	0.81	0.82
Available phosphorus, %	0.48	0.27	0.45	0.25	0.41	0.22

^1^ PC: Positive control diet contained the recommended Ca and non-phytate phosphorus (nPP); NC, negative control diet contained the recommended Ca and low nPP. ^2^ Provided per kg of diet: Vit. A, 12,000 IU; Vit. E, 40 mg; Vit. D_3_, 3000 IU; Vit. K_3_, 3 mg; thiamine, 2 mg; riboflavin, 6 mg; niacin, 45 mg; pantothenic acid, 12 mg; pyridoxine, 5 mg; biotin, 0.075 mg; folic acid, 2 mg; Vit. B_12_, 0.02 mg; manganese, 100 mg; zinc, 60 mg; iron, 30 mg; copper, 10 mg; iodine, 1 mg; selenium, 0.2 mg; cobalt, 0.1 mg.

**Table 2:** Body weight and gain of broiler chickens fed a reduced nPP diet supplemented with phytase, probiotic, *Saccharomyces cerevisiae*, and fumaric acid during the trial.

Treatments ^1^	Body Weight, g						Weight Gain, g					
	IBW	7 d	14 d	21 d	28 d	35 d	1–7 d	8–14 d	15–21 d	22–28 d	29–35 d	Total Gain
PC	43.04	206.26 ^bc^	490.61 ^b^	950.11 ^c^	1557.72 ^c^	2234.99 ^b^	163.22 ^bc^	284.35 ^b^	459.49 ^bc^	607.62 ^b^	677.27 ^a^	2191.95 ^bc^
NC	43.16	201.19 ^c^	449.39 ^b^	890.99 ^d^	1426.49 ^d^	2097.66 ^d^	158.03 ^c^	248.20 ^b^	441.60 ^bc^	535.50 ^c^	671.16 ^a^	2054.50 ^d^
PHY	42.98	211.18 ^ab^	567.30 ^a^	1044.70 ^b^	1684.71 ^b^	2277.92 ^b^	168.20 ^ab^	356.12 ^a^	477.40 ^ab^	640.01 ^b^	593.20 ^b^	2234.93 ^b^
PRO	43.06	215.85 ^a^	606.83 ^a^	1135.52 ^a^	1864.95 ^a^	2394.22 ^a^	172.79 ^a^	390.98 ^a^	528.69 ^a^	729.43 ^a^	529.27 ^b^	2351.16 ^a^
SC	43.18	205.75 ^bc^	578.28 ^a^	999.74 ^b^	1503.57 ^c^	2205.20 ^bc^	162.57 ^bc^	372.53 ^a^	421.46 ^bc^	503.84 ^cd^	701.63 ^a^	2162.02 ^bc^
FA	43.12	204.05 ^c^	479.37 ^b^	890.73 ^d^	1430.32 ^d^	2136.42 ^cd^	160.93 ^c^	275.32 ^b^	411.36 ^c^	539.59 ^d^	706.09 ^a^	2093.30 ^cd^
SEM	0.352	3.211	20.244	24.045	28.880	33.364	3.011	19.616	26.417	38.369	28.050	33.426
*p*-value	0.77	0.003	˂0.001	˂0.001	˂0.001	˂0.001	0.002	˂0.001	0.001	˂0.001	0.005	˂0.001
*Contrast probability* vs. *NC*												
PC	-	0.12	0.05	0.02	0.01	˂0.001	0.21	0.07	0.50	0.02	0.83	0.002
PHY	-	0.003	˂0.001	˂0.001	0.004	˂0.001	0.003	˂0.001	0.18	0.009	0.01	˂0.001
PRO	-	˂0.001	˂0.001	˂0.001	˂0.001	˂0.001	˂0.001	˂0.001	0.002	˂0.001	0.008	˂0.001
SC	-	0.16	˂0.001	˂0.001	0.01	0.002	0.15	˂0.001	0.45	0.41	0.28	0.003
FA	-	0.38	0.15	0.99	0.75	0.25	0.51	0.17	0.26	0.02	0.52	0.26

^a–d^ Means with no common letters within the same column varied significantly at *p* < 0.05. ^1^ PC, positive control diet contained the recommended Ca and nPP; NC, negative control diet contained the recommended level of Ca and low nPP; PHY, NC + 600 U phytase/kg of the diet; PRO, NC + 0.05% multi-strain probiotic; SC, NC + 0.2% *Saccharomyces cerevisiae*; and FA, NC + 0.2% fumaric acid. SEM, standard error of the mean; IBW initial body weight.

**Table 3:** Feed intake and feed conversion ratio (FCR) of broiler chickens fed a reduced nPP diet supplemented with phytase, probiotic, *Saccharomyces cerevisiae*, and fumaric acid during the trial.

Treatments ^1^	Feed Intake, g/Bird/d						FCR, g Feed/g Gain					
	1–7 d	8–14 d	15–21 d	22–28 d	29–35 d	TFI, g	1–7 d	8–14 d	15–21 d	22–28 d	29–35 d	TFCR
PC	26.01 ^ab^	55.02	90.93 ^a^	133.11	171.58 ^ab^	3336.56	1.12 ^b^	1.35 ^b^	1.39 ^ab^	1.53 ^ab^	1.77 ^c^	1.52 ^ab^
NC	27.30 ^a^	56.56	87.44 ^abc^	128.63	175.02 ^ab^	3324.72	1.21 ^a^	1.60 ^b^	1.39 ^ab^	1.68 ^a^	1.83 ^bc^	1.62 ^a^
PHY	23.70 ^cd^	55.90	83.49 ^c^	131.26	169.28 ^b^	3245.41	0.99 ^d^	1.10 ^c^	1.22 ^ab^	1.44 ^ab^	2.00 ^ab^	1.45 ^bc^
PRO	23.19 ^d^	54.81	89.31 ^ab^	132.18	159.64 ^c^	3213.96	0.94 ^d^	0.98 ^c^	1.18 ^b^	1.27 ^b^	2.11 ^a^	1.37 ^c^
SC	24.44 ^cd^	54.94	88.83 ^abc^	127.32	177.36 ^a^	3310.28	1.05 ^c^	1.03 ^c^	1.48 ^a^	1.77 ^a^	1.77 ^c^	1.53 ^ab^
FA	25.07 ^bc^	57.63	84.40 ^bc^	128.53	173.65 ^ab^	3285.02	1.09 ^bc^	1.47 ^a^	1.44 ^a^	1.67 ^a^	1.72 ^c^	1.57 ^ab^
SEM	0.601	2.295	2.348	2.352	3.338	54.48	0.03	0.09	0.10	0.15	0.09	0.06
*p*-value	<0.001	0.78	0.05	0.16	0.003	0.25	<0.001	<0.001	0.05	0.05	0.007	0.01
*Contrast probability* vs. *NC*												
PC	0.06	0.51	0.16	0.08	0.32	0.83	0.007	0.032	0.94	0.34	0.58	0.12
PHY	<0.001	0.78	0.12	0.29	0.11	0.17	<0.001	<0.001	0.15	0.13	0.09	0.01
PRO	<0.001	0.46	0.44	0.16	0.001	0.06	<0.001	<0.001	0.08	0.02	0.009	0.001
SC	<0.001	0.49	0.56	0.59	0.50	0.80	<0.001	<0.001	0.42	0.57	0.56	0.15
FA	0.003	0.65	0.22	0.97	0.67	0.48	<0.001	0.22	0.65	0.92	0.28	0.41

^a–d^ Means with no common letters within the same column varied significantly at *p* < 0.05. ^1^ Treatments’ abbreviations see Table 2. TFI, total feed intake; TFCR, total FCR.

**Table 4:** Carcass yield and relative organ weights of broiler chickens fed a reduced nPP diet supplemented with phytase, probiotic, *Saccharomyces cerevisiae*, and fumaric acid at 35 days of age.

Treatments ^1^	Carcass Traits				Immune Organs		
	Dressing, %	Liver, %	Gizzard, %	Heart, %	Thymus, %	Spleen, %	Bursa of Fabricius, %
PC	77.72 ^ab^	1.98 ^b^	1.54 ^b^	0.42	0.101	0.084 ^c^	0.084 ^c^
NC	75.13 ^bc^	1.95 ^b^	1.57 ^ab^	0.42	0.102	0.065 ^d^	0.069 ^d^
PHY	79.41 ^a^	1.92 ^b^	1.45 ^b^	0.44	0.099	0.088 ^bc^	0.091 ^b^
PRO	79.50 ^a^	1.92 ^b^	1.59 ^ab^	0.43	0.098	0.111 ^a^	0.083 ^c^
SC	76.91 ^ab^	2.01 ^ab^	1.48 ^b^	0.40	0.101	0.109 ^a^	0.098 ^a^
FA	73.38 ^c^	2.07 ^a^	1.68 ^a^	0.42	0.101	0.099 ^ab^	0.101 ^a^
SEM	1.58	0.041	0.069	0.019	0.002	0.007	0.002
*p*-value	0.002	0.008	0.04	0.72	0.83	˂0.001	˂0.001
*Contrast probability* vs. *NC*							
PC	0.11	0.56	0.68	0.90	0.78	0.01	˂0.001
PHY	0.01	0.36	0.11	0.65	0.63	0.002	˂0.001
PRO	0.004	0.46	0.75	0.45	0.44	˂0.001	˂0.001
SC	0.27	0.23	0.22	0.35	0.99	˂0.001	˂0.001
FA	0.28	0.008	0.11	0.78	0.91	˂0.001	˂0.001

^a–d^ Means with no common letters within the same column varied significantly at *p* < 0.05. ^1^ Treatments’ abbreviations see Table 2.

**Table 5:** Nutrient digestibility of broiler chickens fed a reduced nPP diet supplemented with phytase, probiotic, *Saccharomyces cerevisiae*, and fumaric acid at 35 days of age.

Treatments ^1^	Nutrient Digestibility, %					
	DM	CP	EE	CF	Ca	P
PC	67.23 ^b^	78.38 ^c^	51.26 ^cd^	29.85 ^b^	71.46 ^c^	45.82 ^c^
NC	63.71 ^c^	75.81 ^c^	48.57 ^d^	29.28 ^c^	70.18 ^c^	44.02 ^d^
PHY	71.84 ^a^	84.29 ^b^	54.17 ^bc^	30.13 ^b^	79.67 ^a^	51.34 ^a^
PRO	71.85 ^a^	88.44 ^a^	59.54 ^a^	30.80 ^a^	74.56 ^b^	48.91 ^b^
SC	72.87 ^a^	85.77 ^ab^	56.57 ^b^	30.29 ^ab^	76.30 ^b^	50.72 ^a^
FA	72.78 ^a^	84.96 ^b^	51.43 ^cd^	30.19 ^b^	72.07 ^c^	50.01 ^ab^
SEM	1.335	1.492	1.328	0.235	0.824	0.590
*p*-value	˂0.001	˂0.001	˂0.001	0.001	˂0.001	˂0.001
*Contrast probability* vs. *NC*						
PC	0.02	0.11	0.07	0.03	0.15	0.01
PHY	˂0.001	˂0.001	0.001	0.02	˂0.001	˂0.001
PRO	˂0.001	˂0.001	˂0.001	0.002	0.003	˂0.001
SC	˂0.001	˂0.001	˂0.001	0.008	0.002	˂0.001
FA	˂0.001	˂0.001	0.05	0.02	0.07	˂0.001

^a–d^ Means with no common letters within the same column varied significantly at *p* < 0.05.^1^ Treatment abbreviations see Table 2. DM, dry matter; CP, crude protein; EE, ether extract; CF, crude fiber; Ca, calcium; P, phosphorus.

**Table 6:** Serum constituents and hormone profile of broiler chickens fed a reduced nPP diet supplemented with phytase, probiotic, *Saccharomyces cerevisiae,* and fumaric acid at 21 days of age.

Treatments ^1^	TC, mg/dL	TG, mg/dL	Glucose, mg/dL	Ca, mg/dL	P, mg/dL	T3, ng/mL	T4, ng/mL	ALP, U/L
PC	136.57 ^a^	129.55 ^a^	246.72 ^a^	11.78 ^b^	5.36 ^a^	1.88 ^bc^	13.16 ^b^	36.92
NC	127.73 ^b^	129.06 ^a^	247.28 ^a^	10.19 ^d^	5.01 ^b^	1.79 ^c^	11.02 ^c^	36.95
PHY	122.56 ^c^	97.83 ^e^	242.63 ^b^	11.75 ^b^	5.36 ^a^	2.00 ^ab^	13.66 ^b^	37.00
PRO	128.02 ^b^	90.06 ^d^	240.06 ^b^	11.96 ^a^	5.38 ^a^	2.03 ^a^	14.29 ^a^	36.90
SC	121.97 ^c^	103.90 ^c^	242.21 ^b^	11.61 ^bc^	5.36 ^a^	1.75 ^c^	13.45 ^b^	36.78
FA	123.95 ^c^	107.55 ^b^	243.33 ^b^	11.47 ^c^	5.33 ^a^	2.00 ^ab^	13.60 ^b^	36.47
SEM	1.285	0.720	1.50	0.085	0.065	0.065	0.30	0.735
*p*-value	˂0.001	˂0.001	0.002	0.003	0.002	˂0.001	˂0.001	0.98
*Contrast probability* vs. *NC*								
PC	˂0.001	0.88	0.71	˂0.001	0.01	0.18	˂0.001	0.97
PHY	˂0.001	˂0.001	0.01	˂0.001	0.01	0.004	˂0.001	0.95
PRO	0.82	˂0.001	0.005	˂0.001	˂0.001	0.001	˂0.001	0.94
SC	˂0.001	˂0.001	0.008	˂0.001	0.01	0.54	˂0.001	0.82
FA	0.007	0.01	0.02	0.003	0.002	0.004	˂0.001	0.52

^a–e^ Means with no common letters within the same column varied significantly at *p* < 0.05.^1^ Treatment abbreviations, see Table 2. TC, total cholesterol; TG, triglyceride; Ca, calcium; P, phosphorus; T3, triiodothyronine; T4, thyroxine; ALP, alkaline phosphatase.

**Table 7:** Serum constituents and hormone profile of broiler chickens fed a reduced nPP diet supplemented with phytase, probiotic, *Saccharomyces cerevisiae*, and fumaric acid at 35 days of age.

Treatments ^1^	TC, mg/dL	TG, mg/dL	Glucose, mg/dL	Ca, mg/dL	P, mg/dL	T3, ng/mL	T4, ng/mL	ALP, U/L
PC	161.97 ^b^	132.55 ^b^	235.60 ^ab^	11.32 ^b^	5.35 ^a^	2.60 ^a^	13.09 ^c^	30.16 ^b^
NC	167.93 ^a^	139.69 ^a^	238.19 ^a^	10.67 ^c^	5.02 ^b^	2.18 ^d^	13.15 ^c^	40.43 ^a^
PHY	138.66 ^d^	125.02 ^c^	231.76 ^c^	11.85 ^a^	5.40 ^a^	2.45 ^bc^	14.73 ^a^	32.74 ^b^
PRO	130.07 ^e^	119.11 ^d^	232.52 ^bc^	11.76 ^a^	5.43 ^a^	2.43 ^c^	14.25 ^ab^	33.11 ^b^
SC	142.69 ^cd^	119.98 ^d^	236.80 ^a^	11.87 ^a^	5.42 ^a^	2.19 ^d^	14.06 ^b^	33.23 ^b^
FA	145.77 ^c^	129.53 ^b^	238.06 ^a^	11.58 ^b^	5.40 ^a^	2.56 ^ab^	13.79 ^b^	33.46 ^b^
SEM	1.450	1.110	1.60	0.095	0.076	0.055	0.285	0.900
*p*-value	˂0.001	˂0.001	0.001	˂0.001	˂0.001	˂0.001	˂0.001	˂0.001
*Contrasts probability* vs. *NC*								
PC	˂0.001	˂0.001	0.12	˂0.001	˂0.001	˂0.001	0.84	˂0.001
PHY	˂0.001	˂0.001	0.001	˂0.001	˂0.001	0.01	˂0.001	˂0.001
PRO	˂0.001	˂0.001	0.002	˂0.001	˂0.001	0.005	0.001	˂0.001
SC	˂0.001	˂0.001	0.39	˂0.001	˂0.001	0.47	0.004	˂0.001
FA	˂0.001	˂0.001	0.94	˂0.001	˂0.001	˂0.001	0.03	˂0.001

^a–d^ Means with no common letters within the same column varied significantly at *p* < 0.05. ^1^ Treatment abbreviations, see Table 2. TC, total cholesterol; TG, triglyceride; Ca, calcium; P, phosphorus; T3, triiodothyronine; T4, thyroxine; ALP, alkaline phosphatase.

**Table 8:** Tibia physical traits and mineralization of broiler chickens fed a reduced nPP diet supplemented with phytase, probiotic, *Saccharomyces cerevisiae*, and fumaric acid at 35 days of age.

	Tibia Physical Characteristics				Tibia Chemical Composition				
Treatments ^1^	Dry Weight, g	Length, mm	Width, mm	TBS, kg/cm^2^	DM, %	Ash, % (Fat-Free Dry Basis)	Ca, % (Fat-Free Dry Tibia Weight)	P, % (Fat-Free Dry Tibia Weight)	Ca:P
PC	5.60 ^c^	105.47 ^c^	7.21 ^b^	22.16 ^c^	66.76	41.73 ^d^	32.31 ^c^	16.05 ^c^	2.02 ^bc^
NC	5.30 ^d^	100.96 ^d^	6.97 ^c^	17.91 ^d^	64.60	38.43 ^e^	30.87 ^c^	15.13 ^d^	2.04 ^abc^
PHY	7.07 ^a^	122.31 ^a^	7.25 ^b^	27.06 ^b^	69.18	45.57 ^c^	37.26 ^a^	17.58 ^b^	2.12 ^ab^
PRO	7.04 ^a^	109.26 ^b^	7.30 ^b^	30.62 ^a^	70.77	47.27 ^b^	37.77 ^a^	18.43 ^a^	2.05 ^abc^
SC	6.26 ^b^	111.14 ^b^	7.72 ^a^	26.61 ^b^	65.41	49.93 ^a^	34.31 ^b^	17.53 ^b^	1.96 ^c^
FA	5.69 ^c^	105.72 ^c^	7.58 ^a^	21.90 ^c^	69.26	40.94 ^d^	35.09 ^b^	16.30 ^c^	2.15 ^a^
SEM	0.127	1.513	0.113	1.074	2.979	0.687	0.764	0.16	0.052
*p*-value	0.001	0.002	˂0.001	˂0.001	0.29	˂0.001	˂0.001	˂0.001	0.02
*Contrast probability* vs. *NC*									
PC	0.03	0.01	0.04	0.001	0.48	˂0.001	0.08	˂0.001	0.63
PHY	0.001	0.001	0.03	˂0.001	0.14	˂0.001	˂0.001	˂0.001	0.15
PRO	0.001	0.005	0.01	˂0.001	0.05	˂0.001	˂0.001	˂0.001	0.86
SC	0.007	0.003	˂0.001	˂0.001	0.78	˂0.001	˂0.001	˂0.001	0.13
FA	0.01	0.01	˂0.001	0.002	0.14	0.002	˂0.001	˂0.001	0.05

^a–d^ Means with no common letters within the same column varied significantly at *p* < 0.05. ^1^ Treatment abbreviations, see Table 2. TBS, tibia breaking strength; DM, dry matter; Ca, calcium; P, phosphorus.

**Table 9:** Small intestine histomorphology of broiler chickens fed a reduced nPP diet supplemented with phytase, probiotic, *Saccharomyces cerevisiae*, and fumaric acid at 35 days of age.

Treatments ^1^	VH, µm	VW, µm	CD, µm	MT, µm	VH/CD	VSA, µm^3^
	Duodenum					
PC	1685.67 ^ab^***	280.00 ^a^***	275.33 ^abc^	263.67 ^a^**	6.12 ^a^**	1.48 ^a^***
NC	1422.67 ^c^	210.00 ^c^	266.00 ^c^	241.00 ^b^	5.35 ^b^	0.94 ^c^
PHY	1656.67 ^ab^***	266.00 ^ab^***	286.33 ^a^*	263.33 ^a^**	5.79 ^ab^	1.39 ^ab^***
PRO	1706.00 ^a^***	267.33 ^ab^***	280.67 ^ab^	262.67 ^a^**	6.08 ^a^**	1.43 ^a^***
SC	1630.67 ^b^***	249.67 ^b^**	268.33 ^bc,^*	263.33 ^a^**	6.08 ^a^**	1.28 ^b^***
FA	1694.33 ^ab^***	260.00 ^ab^***	274.00 ^abc^	272.00 ^a^**	6.19 ^a^**	1.38 ^ab^***
SEM	27.306	9.497	5.699	4.526	0.181	0.054
*p*-value	˂0.001	˂0.001	0.03	0.003	0.005	˂0.001
Jejunum						
PC	1390.33 ***	260.33 ^b^***	236.00 ^a^**	291.33 ^a^***	5.89 ^b^*	1.14 ^b^***
NC	1216.33 ^b^	222.00 ^c^	183.33 ^b^	247.67 ^d^	6.64 ^a^	0.85 ^c^
PHY	1458.67 ^a^***	278.33 ^a^***	239.67 ^a^**	280.33 ^ab^***	6.09 ^ab^	1.28 ^a^***
PRO	1461.67 ^a^***	281.67 ^a^***	242.33 ^a^**	270.00 ^bc^***	6.04 ^ab^	1.29 ^a^***
SC	1447.00 ^a^***	272.33 ^ab^***	236.67 ^a^**	271.00 ^bc^***	6.11 ^ab^	1.24 ^a^***
FA	1430.67 ^a^***	274.67 ^ab^***	244.00 ^a^**	264.67 ^c^**	5.87 ^b^*	1.23 ^a^***
SEM	31.071	6.656	7.694	4.401	0.204	0.042
*p*-value	˂0.001	˂0.001	0.002	˂0.001	0.03	˂0.001
Ileum						
PC	898.33 ^a^**	285.09 ^ab^***	261.33 ^a^***	271.33 ^a^**	3.44 ^bc^*	0.80 ^a^***
NC	825.33 ^c^	246.41 ^d^	188.33 ^c^	230.67 ^b^	4.39 ^a^	0.64 ^c^
PHY	864.67 ^b^**	277.16 ^bc^***	242.67 ^b^***	265.33 ^a^*	3.57 ^b^*	0.75 ^b^***
PRO	864.33 ^b^**	273.01 ^c^**	244.67 ^b^***	256.00 ^a^*	3.54 ^b^*	0.74 ^b^***
SC	879.33 ^a b^**	295.58 ^a^***	268.33 ^a^***	257.00 ^a^**	3.28 ^bc^*	0.82 ^a^***
FA	866.33 ^b^**	275.79 ^bc^**	274.33 ^a^***	261.33 ^a^**	3.16 ^c^*	0.75 ^b^***
SEM	11.657	4.899	7.11	8.221	0.143	0.013
*p*-value	0.001	˂0.001	˂0.001	0.006	0.01	˂0.001

^a–d^ Means with no common letters within the same column varied significantly at *p* < 0.05. *, *p* < 0.05; **, *p* < 0.01; ***, *p* < 0.001 probability of contrasts vs. NC. ^1^ Treatment abbreviations, see Table 2. VH, villi height; VW, villi width; CD, crypt depth; MT, muscular thickness; VSA, villi absorptive surface area.

## Data Availability

The data presented in this study are available on request from the corresponding author.
